# How close is autophagy-targeting therapy for Alzheimer's disease to clinical use? A summary of autophagy modulators in clinical studies

**DOI:** 10.3389/fcell.2024.1520949

**Published:** 2025-01-08

**Authors:** Sofia Miranda Fernandes, Johanna Mayer, Per Nilsson, Makoto Shimozawa

**Affiliations:** Department of Neurobiology, Care Sciences and Society, Division of Neurogeriatrics, Center for Alzheimer Research, Karolinska Institutet, Stockholm, Sweden

**Keywords:** Alzheimer’s disease, protein homeostasis, autophagy modulators, clinical studies, autophagy impairment

## Abstract

Alzheimer’s disease (AD) is a neurodegenerative disorder clinically characterized by progressive decline of memory and cognitive functions, and it is the leading cause of dementia accounting for 60%–80% of dementia patients. A pathological hallmark of AD is the accumulation of aberrant protein/peptide aggregates such as extracellular amyloid plaques containing amyloid-beta peptides and intracellular neurofibrillary tangles composed of hyperphosphorylated tau. These aggregates result from the failure of the proteostasis network, which encompasses protein synthesis, folding, and degradation processes. Autophagy is an intracellular self-digesting system responsible for the degradation of protein aggregates and damaged organelles. Impaired autophagy is observed in most neurodegenerative disorders, indicating the link between autophagy dysfunction and these diseases. A massive accumulation of autophagic vacuoles in neurons in Alzheimer’s brains evidences autophagy impairment in AD. Modulating autophagy has been proposed as a therapeutic strategy for AD because of its potential to clear aggregated proteins. However, autophagy modulation therapy for AD is not yet clinically available. This mini-review aims to summarize clinical studies testing potential autophagy modulators for AD and to evaluate their proximity to clinical use. We accessed clinicaltrials.gov provided by the United States National Institutes of Health to identify completed and ongoing clinical trials. Additionally, we discuss the limitations and challenges of these therapies.

## 1 Introduction

Alzheimer’s disease (AD) is a neurodegenerative disorder clinically characterized by progressive impairment of memory and cognitive functions (Panza et al., 2019; Knopman et al., 2021). AD is the most common cause of dementia accounting for 60%–80% of dementia patients (Alzheimer’s disease facts and figures, 2024). The number of patients with dementia has been increasing over the years, which is estimated to be more than 55 million people worldwide today and 150 million people by 2050 (Collaborators, 2022). AD is pathologically characterized by the accumulation of aberrant protein and peptide aggregation including extracellular amyloid plaques containing amyloid-beta (Aβ) peptides and intracellular neurofibrillary tangles (NFTs) composed of hyperphosphorylated tau. Aβ peptides are secreted to the extracellular space as a consequence of sequential enzymatic cleavages of a type-1 membrane protein, amyloid precursor protein (APP), by β- and γ-secretases (Panza et al., 2019). Tau is a microtubule-associated protein that stabilizes microtubules in the axons of neurons under physiological conditions. In pathological conditions, tau undergoes abnormal posttranslational modifications, such as acetylation and hyperphosphorylation, leading to the formation of aggregated tau (Knopman et al., 2021). These protein aggregates are caused by the failure of the proteostasis network, consisting of protein synthesis, folding, and degradation processes.

**FIGURE 1:**
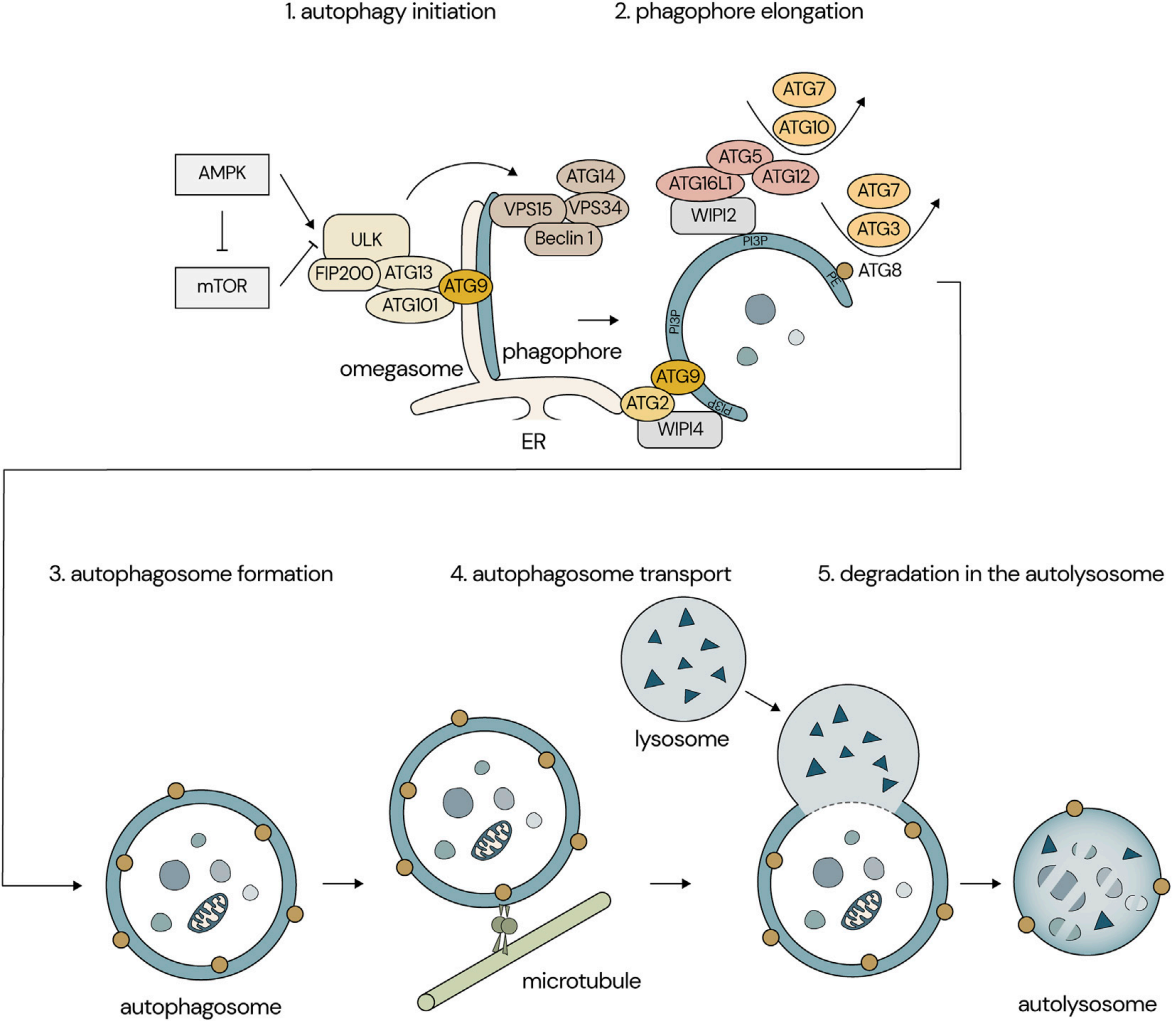
Overview of the autophagic process in neurons. (1) Several signaling pathways, including mTOR and AMPK, regulate the ULK1 complex, which initiates membrane nucleation and phagophore formation. (2, 3) The phagophore elongates into a closed double-membranous structure called an autophagosome. (4) The autophagosome is transported along microtubules to the neuronal soma. (5) The autophagosome fuses with the lysosome, paving the way for the degradation of the autophagosomal content.

Proteasomes and lysosomes are the two major protein-degrading systems in the cells. In general, proteasomes degrade short-lived and soluble misfolded proteins, whereas lysosomes degrade long-lived and insoluble aggregated proteins (Zhao et al., 2022). Autophagy is a part of the lysosomal degrading system and is impaired in most neurodegenerative disorders including AD. Autophagy is categorized into three diverse types: microautophagy, macroautophagy, and chaperone-mediated autophagy (CMA) (Wang et al., 2023). Microautophagy is a process which incorporates cytosolic components into late endosomes or lysosomes by invagination (Kuchitsu and Taguchi, 2024). Macroautophagy starts with the formation of a double membrane vacuole called autophagosome. Cytosolic components including aggregated proteins and damaged organelles are sequestered by the autophagosome and degraded by fusion of the autophagosome and lysosome (Figure 1). In CMA, cytosolic proteins with KFERQ-like motifs (Lys-Phe-Glu-Arg-Gln) bind a chaperone protein, the heat shock cognate protein of 71 kDa (Hsc70). Target proteins are unfolded and taken into the lysosomal lumen through a multimeric complex known as lysosome-associated membrane protein 2A (LAMP2A) (Valdor and Martinez-Vicente, 2024; Kaushik and Cuervo, 2018). Macroautophagy is the most well-studied type of autophagy in the context of AD, hence we will focus on macroautophagy and is hereafter referred to as autophagy. Neurons have a unique autophagic process because of their highly polarized cell shape. Autophagosomes are formed not only in neuronal cell bodies but also in distal axons. Thus, autophagosomes need to be transported on microtubules along axons to the soma by the dynein-dynactin motor machinery (Hill and Colón-Ramos, 2020) (Figure 1). During its retrograde transport, autophagosomes fuse with late endosomes to form amphisomes and with lysosomes to form autolysosomes, leading to the gradual acidification of autophagosomes. Non-dividing cells including neurons are heavily dependent on autophagy for protein homeostasis because these cells cannot dilute toxic aggregates by proliferation as opposed to dividing cells. In addition, synapses are extremely dynamic structures requiring a constant remodelling that involves both protein degradation including autophagy and synthesis of new proteins (Bai et al., 2024).

## 2 Autophagy impairment in Alzheimer's disease

In 1964, several electron microscopy studies firstly described an accumulation of dense bodies around Aβ plaques in AD brain (Bubis and Luse, 1964; Terry et al., 1964; Kidd, 1964). Following this finding, extensive research has revealed a significant impairment of autophagy in AD, indicated by an accumulation of autophagic vacuoles (AVs) in neurons in both postmortem AD brains and AD mouse model brains. (Nixon et al., 2005; Yu et al., 2005; Jiang et al., 2022; Naia et al., 2023). Three different causes of autophagy impairment in AD are proposed so far; 1) altered autophagy initiation, 2) inhibition of AV transport, and 3) impairment of AV degradation caused by lysosomal dysfunction. The impairment of autophagy is most likely caused by a combination of these mechanisms, and the alteration of the autophagy system may differ depending on the stage of the disease.

### 2.1 Altered autophagy initiation

The expression of Beclin-1, a component of one of the autophagy initiator complexes (Figure 1), is downregulated in moderate to severe AD brains (Pickford et al., 2008). In addition, a reduction of mRNA levels of core autophagy machinery for autophagosome formation including the Beclin-1 gene (*BECN1)* was reported in the hippocampus of AD brains, suggesting decreased initiation of autophagy as an explanation of autophagy inhibition in AD brains (Lachance et al., 2019). Recently, Tumurbaatar et al., revealed that the protein levels of autophagy-related genes (ATGs) including Beclin-1 are reduced in postmortem hippocampal samples of AD patients, whereas these protein levels are preserved in non-demented individuals with AD neuropathology (Tumurbaatar et al., 2023). On the other hand, others reported that negative and positive regulators of autophagy flux are downregulated and upregulated, respectively, in the entorhinal cortex of AD patients (Lipinski et al., 2010). Concordantly, an increased autophagy initiation in hippocampal neurons of AD brains was also reported (Bordi et al., 2016). Thus, the alteration of autophagy initiation in AD brains is still under discussion.

### 2.2 Inhibition of AV transport

A disruption of the interaction between amphisomes and dynein complexes was shown in an AD transgenic mouse model (Tammineni et al., 2017). Aβ oligomers interact with amphisomes and inhibit the recruitment of amphisomes by dynein, which leads to its accumulation in axons. Since tau is involved in the stabilization of microtubules and axonal transport under physiological conditions, a destabilization of microtubules caused by dissociation of hyperphosphorylated tau may also lead to the impairment of autophagosome transport in AD (Wang et al., 2015).

### 2.3 Impairment of AV degradation

Lysosomal dysfunction has been suggested in human AD brains and the brains of AD mouse models. An early upregulation of lysosomal protein cathepsin D levels is observed in AD brains (Cataldo et al., 1995). Presenilin-1 (PS1) is the catalytic subunit of γ-secretase which is the key enzyme in Aβ production from APP. Mutations have been found in more than 100 sites of PS1 in familial AD (FAD), which may increase the Aβ42 to Aβ40 ratio and lead to Aβ plaque depositions. In addition to the role of PS1 in Aβ plaque formation, Lee JH et al., showed its essential function for lysosomal acidification (Lee et al., 2015). Recently, the same group revealed that failed acidification of autolysosomes caused by lowered vATPase activity leads to autolysosome accumulation in AD transgenic mouse models (Lee et al., 2022).

## 3 Autophagy-targeting therapy for Alzheimer's disease

Since autophagy is severely impaired in AD and plays an important role in aggregated protein clearance, the modulation of autophagic activities has been proposed as therapeutics for AD (Menzies et al., 2017; Liu and Li, 2019; Festa et al., 2021; Zhang et al., 2021). In this review, we will focus on autophagy-targeting AD therapies under evaluation in clinical studies to summarize how close autophagy-targeting therapies are to clinical use. We used ClinicalTrials.gov, a clinical research registry provided by the United States National Institutes of Health, to identify completed and ongoing clinical trials with potential autophagy modulators for AD therapies.

### 3.1 The mammalian target of rapamycin inhibitors

The mammalian target of rapamycin (mTOR) inhibitors including rapamycin, and its analogues are the most intensively investigated autophagy activators. mTOR consists of two different complexes, mTOR complex 1 (mTORC1) and complex 2 (mTORC2). The mTORC1 suppresses autophagy through the inhibition of unc-51-like autophagy activating kinase 1 (ULK1) complex which is a key enzyme complex for autophagy initiation in cells (Figure 1), and therefore inhibition of mTORC1 induces activation of autophagy (Panwar et al., 2023). Rapamycin supplemented in food improved memory function, and reduced Aβ42 and phosphorylated tau levels in AD mouse models at an early stage of pathology development. On the other hand, these effects were no longer observed when rapamycin was administered at a later stage characterized by prominent Aβ plaques and tau tangles (Caccamo et al., 2010; Spilman et al., 2010; Majumder et al., 2011). One early Phase I study was performed to measure rapamycin concentration in the cerebrospinal fluid (CSF) and evaluate the feasibility and safety of rapamycin treatment in a small number of mild cognitive impairment (MCI) or early AD patients (NCT04200911, Table 1). Sixteen adverse events are reported, including one serious adverse event, a possible transient ischemic attack (ITA), which was however unrelated to the study intervention. Two clinical trials of rapamycin treatment for AD are ongoing (NCT04629495, NCT06022068). These are Phase II and Phase I/II studies targeting amnestic MCI (aMCI) and early-stage AD.

**TABLE 1:** Summary of autophagy targeting therapy for AD.

Compound	Subject number	Length of study	Level/dosage	Condition	Outcomes	Clinical trial/References
*mTOR inhibitor*						
Rapamune (Sirolimus)	10	8 weeks	1 mg/day	MCI and EAD	-No penetration in the CSF -One serious adverse event (unrelated to study intervention)	NCT04200911
Rapamycin (Sirolimus)	15	6 months	7 mg/week	MCI and EAD	Ongoing studies (estimated completion date 2025)	NCT06022068
Rapamycin	40	A 12-month treatment period followed by a post-treatment assessment (14-days after) and a final assessment (6-months after)	1 mg/day	aMCI and EAD	Ongoing studies (estimated completion date 2026)	NCT04629495
*AMPK activator*						
Metformin	80	12 months	2,000 mg/day	aMCI with overweight (non-diabetic)	-Better SRT scores for memory (Luchsinger et al., 2016)	NCT00620191
Metformin	20	An 8-week treatment period and an 8-week placebo period (crossover)	ascending dose from 500–2,000 mg/day	MCI or early dementia due to AD (non-diabetic)	-Ameliorated executive functioning -Improved overall learning/memory and attention (Koenig et al., 2017)	NCT01965756
Metformin	326	18 months	ascending dose from 500–2,000 mg/day	Early and late aMCI	Ongoing studies (estimated completion date 2027)	NCT04098666
Trehalose (Mycose)	20	12 weeks	15 g/week	AD	Unknown status	NCT04663854
Metformin combined with lifestyle-based intervention	600	24 months	ascending dose from 500–1,000 mg/day or 2000 mg/day	Older people with risk factors for dementia	Ongoing studies (estimated completion date 2027)	NCT05109169
*Abl tyrosine kinase inhibitor*						
Nilotinib	37	52 weeks	150 mg/day (6 months) followed by 300 mg/day (6 months)	Mild to moderate AD	-Good overall tolerability -Attenuated amyloid accumulation in the frontal lobe -Decreased CSF Aβ40 (6-month) and Aβ42 levels (12-month) -Reduced P-tau 181 (6- and 12-month) -Attenuated hippocampal volume loss (Turner et al., 2020)	NCT02947893
Nilotinib BE	1,275	72 weeks	84 or 112 mg/day	EAD	Ongoing studies (estimated completion date 2026)	NCT05143528
*IMPase and GSK-3β inhibitors*						
Lithium and Divalproex	35	6 weeks	-	AD	Completed. No results posted	NCT00088387
Lithium Carbonate	45	12 months (Interim analysis)	0.25–0.5 mmol/L	aMCI	-Decreased CSF P-tau levels -Better performance on the cognitive subscale of the Alzheimer’s Disease Assessment Scale and in attention tasks -Good overall tolerability (Forlenza et al., 2011)	NCT01055392
Lithium Carbonate	61	24-month treatment period and 24-month follow-up period	0.25–0.5 mEq/L	aMCI	-Attenuated cognitive and functional decline (24-month) -Increased CSF Aβ_1-42_ levels (36-month) (Forlenza et al., 2019)	NCT01055392
Lithium	77	12 weeks	150–600 mg/day	AD with agitation	-Did not have any effect on the treatment of agitation/aggression -Improvement of Clinical Global Impression (CGI) score and excellent safety (Devanand et al., 2022)	NCT02129348
Lithia water combined with TMS	100	A 4-week treatment period and a 4-week placebo period (crossover)	Lithia water (diet supplementary)	AD	Unknown status	NCT02204969
Lithium Carbonate	80	2 years	0.5–0.8 mEq/L	MCI due to AD	Completed. No results posted	NCT03185208
AL001 (lithium salicylate)	72	A 14-day treatment period, and a 42-day follow-up period	9 cohorts multiple ascending dose	Mild to moderate AD	Completed. No results posted	NCT05363293
NanoLithium® NP03	68	Double blind 12-week -period, followed by an open-label 36-week period	1.8 mg/day	Mild-to-severe AD	Ongoing studies (estimated completion date 2025)	NCT05423522
*Monoclonal antibody to sortilin (SORT1)*						
AL101 (GSK-4527226)	282	18 months	2 dose levels of intravenous infusions	EAD (MCI and mild dementia due to AD)	Ongoing studies (estimated completion date 2029)	NCT06079190

### 3.2 Adenosine monophosphate-activated protein kinase activators

Adenosine monophosphate-activated protein kinase (AMPK) is another autophagy regulator thoroughly investigated. AMPK induces autophagy by direct phosphorylation of ULK1. AMPK also negatively regulates mTOR activity, leading to the activation of autophagy (Figure 1). Metformin and resveratrol are AMPK activators and their potential for AD therapy has been assessed in AD mouse models such as APP/PS1 mice (Chen et al., 2021; Vingtdeux et al., 2010; Zhao et al., 2023). These experiments using AD mouse models showed that AMPK activators reduced Aβ and tau pathologies. Metformin is used as a treatment for type 2 Diabetes Mellitus and is under investigation for AD prevention. Two small Phase II studies of metformin were performed in MCI or mild dementia AD patients (NCT00620191, NCT01965756). In the study conducted at Columbia University, the metformin-treated group showed significantly greater improvement in the score of the Selective Reminding Test for memory compared with the placebo group (Luchsinger et al., 2016). The second clinical study found a significantly better score in the Trails B executive function test by metformin treatment compared to placebo (Koenig et al., 2017). Currently, the placebo-controlled Phase II/III study is ongoing in early or late MCI subjects (NCT04098666). In addition, a Phase II study is recruiting elderly adults at risk for dementia to assess the preventive effects of a combination of metformin and lifestyle intervention on changes in cognition (NCT05109169) (Barbera et al., 2024). Although metformin induces autophagy through the activation of AMPK, it is unclear to what extent autophagy activation contributes to the improvement of memory functions due to additional effects of metformin including modulation of BACE1 expression levels, neuroinflammation, and glucose metabolism (Liao et al., 2022). It is worth noting that a recent study using an AD mouse model showed that metformin treatment increased Aβ plaque formation and phospho-tau levels and caused memory impairment (Cho et al., 2024). Therefore, further assessment of metformin for AD treatment is needed. The disaccharide trehalose potentially activates autophagy through AMPK-dependent and independent mechanisms. Trehalose mimics a starvation condition by blocking the glucose transporter family, leading to the activation of AMPK (Mardones et al., 2016; DeBosch et al., 2016). Trehalose also induces the nuclear translocation of transcription factor EB, which is a central regulator of autophagic-lysosomal protein expression (Rusmini et al., 2019). Inconsistently, some *in vitro* studies using cell models showed that trehalose inhibits the autophagosome degradation step rather than activating autophagy, raising the question of whether trehalose is an autophagy activator (Yoon et al., 2017; Tien et al., 2016). Although data from different *in vitro* studies have shown controversial effects of trehalose on autophagy, studies using AD animal models showed that trehalose treatment reduced Aβ levels and tau aggregation and ameliorated cognitive impairment, suggesting neuroprotective effects of trehalose (Perucho et al., 2012; Schaeffer et al., 2012; Du et al., 2013; Portbury et al., 2017). A randomized Phase I study was conducted to evaluate the effectiveness of trehalose in reducing AD symptoms with 20 AD patients (NTC04663854).

### 3.3 Nilotinib

Nilotinib is a tyrosine kinase inhibitor approved for the treatment of chronic myelogenous leukaemia by the FDA. Nilotinib selectively inhibits the autophosphorylation of Bcr-Abl, which increases parkin-Beclin-1 interaction leading to autophagic vacuole clearance. In addition, nilotinib successfully reduced Aβ plaque load in Tg-SwDI mice (Lonskaya et al., 2013; Lonskaya et al., 2014). A small Phase II study was conducted to assess the safety and tolerability of nilotinib and changes in AD biomarkers in mild to moderate AD patients (NTC02947893). Aβ40 and Aβ42 levels in the CSF were reduced in the nilotinib-treated group compared to the placebo group at 6- and 12-months, respectively. Additionally, phospho-tau 181 levels were reduced in the nilotinib-treated group at 6- and 12-months (Turner et al., 2020). Currently, the efficacy and safety of nilotinib bioequivalent are being evaluated in a Phase III study in early AD patients (NTC05143528).

### 3.4 Lithium

Lithium is also a potential autophagy-activating medication which has already been approved for the treatment of manic episodes and bipolar disorder by the FDA (Shen et al., 2024). Li^+^ replaces Mg^2+^ at the binding site of Mg^2+^-dependent enzymes, thus Li^+^ inhibits the activity of multiple enzymes including inositol monophosphatase (IMPase), Akt/b-arrestin-2, and glycogen synthase kinase-3β (GSK-3β) (Puglisi-Allegra et al., 2023). Autophagy is activated through the inhibition of IMPase leading to the reduction of free inositol-1, 4, 5-triphosphate (IP3) levels (Sarkar et al., 2005). Multiple clinical studies of lithium have been conducted to assess its effect on memory and cognitive function as well as on behavioural and psychiatric symptoms of dementia (BPSD) in AD (Table 1). Despite that several clinical studies have shown beneficial effects of lithium on AD and aMCI patients, there is a limitation of lithium usage especially in elderly people because of its narrow therapeutic index, and high risk of side effects and toxicity (Murphy et al., 2023). To overcome this problem, currently, two new types of lithium medication for AD are under evaluation in clinical studies. NanoLithium is composed of lithium citrate and a water-in-oil microemulsion drug delivery system, being transported by lipoproteins into the cytoplasm of cells through lipoprotein receptors. By using this drug delivery system, NanoLithium achieved pharmacological activity at lower doses compared with classical lithium treatments (Guilliot et al., 2024). In animal model studies, NanoLithium rescued memory function in early- and late-stage AD rat models (Guilliot et al., 2024; Wilson et al., 2017). A Phase II proof-of-concept clinical study is being conducted since 2022 (NCT05423522). AL001 is another approach of lithium delivery by using ionic cocrystals of lithium salicylate with organic anions. The safety and maximum tolerated dose of AL001 are under evaluation in a Phase I/IIa study (NCT05363293). The previous study was completed in 2023, but the results have not been posted on ClinicalTrials.gov yet.

### 3.5 A monoclonal antibody to improve lysosomal function

As previously mentioned, lysosomal dysfunction in AD brains is one of the causes of autophagy impairment. Thus, the improvement of lysosomal function is another approach for autophagy activation. Progranulin (PGRN) is a secreted protein acting as an autocrine and paracrine neurotrophic factor which regulates neuronal survival, axonal growth, and neuroinflammation (Rhinn et al., 2022; Boylan et al., 2023). PGRN also regulates lysosomal function by acting as a chaperone for lysosomal enzymes including cathepsin D. Heterozygous loss-of-function PGRN gene (*GRN*) mutations cause frontotemporal lobar degeneration (FTLD) with accumulation of transactivation response DNA-binding protein 43 (TDP-43) by reducing more than 50% of PGRN protein levels. In addition, a link between PGRN and AD has been suggested: several mutations in *GRN* were found in AD patients (Kelley et al., 2010; Cortini et al., 2008; Brouwers et al., 2007). A meta-analysis showed an increased risk of AD associated with the single-nucleotide polymorphism (SNP) rs5848 in the *GRN* gene, which leads to a reduction in PGRN protein levels in the plasma, CSF, and brain tissue (Nicholson et al., 2014; Rademakers et al., 2008). PGRN is degraded via entering the lysosome via its clearance receptor, sortilin. GSK4527226, a monoclonal antibody against sortilin, blocks the binding between sortilin and PGRN which increases PGRN levels in the brain. A Phase II study evaluating the efficacy and safety of GSK4527226 is ongoing in early AD patients (NCT06079190).

## 4 Discussion

Since autophagy is involved in both Aβ and tau metabolism and has close bearings to neurodegeneration, it may serve as a potential therapeutic target for AD. Although several potential autophagy modulators are under evaluation in clinical studies for MCI and AD patients, the specificity of these treatments remains unclear. For example, mTOR is a protein kinase contributing to multiple cellular functions including glucose and lipid metabolism, nucleotide biogenesis, and protein biogenesis (Panwar et al., 2023). In addition, targeting such a multifunctional protein with a small compound which can affect the whole body may cause unfavourable effects. This risk could be reduced with drug delivery systems that pass the blood-brain barrier (BBB) and target specific cells including neurons and microglia, hence increasing therapeutic efficacy and leading to lower exposure to other organs (Wu et al., 2023; Guo et al., 2020). Another challenge for the development of autophagy modulators is the lack of autophagy biomarkers evaluating autophagy activity *in vivo*. Currently available autophagy assays for *in vitro* and *in vivo* research are based on direct detection of autophagic proteins in tissues by using antibodies or expression of exogenous autophagy flux reporter genes such as RFP-GFP-LC3 (Mizushima and Murphy, 2020). However, none of them are suitable for detecting autophagy activity in human brains. Without reliable biomarkers to evaluate autophagy activity in the human brain, the development of AD therapeutics targeting autophagy is impossible. A positron emission tomography (PET) tracer detecting a specific autophagy substrate could be an approach for autophagy biomarkers (Mizushima and Murphy, 2020). Another possibility is the detection of autophagic proteins in the CSF. Changes in autophagic protein levels, such as LC3 and p62, have been reported in CSF from patients with neurodegenerative disorders (Youn et al., 2018; Rubino et al., 2022). However, the relationship between these protein levels in the CSF and autophagy activity in the brain remains unclear. Extensive research has revealed a significant impairment of autophagy in AD and highlighted the importance of functional autophagy in clearing aggregated proteins, making autophagy a promising target for AD therapeutics. However, it is still unclear how much autophagy activation contributes to the therapeutic effect in the therapies currently being investigated in clinical trials, because most treatments have multiple mode of actions. Identifying autophagy modulators with higher specificity, i.e., compounds that specifically target the different steps of autophagy; initiation, transport and clearance, and biomarkers assessing autophagy activation *in vivo* may accelerate the development of autophagy targeting therapeutics for AD.
